# Quorum Sensing Inhibitors from the Sea Discovered Using Bacterial *N*-acyl-homoserine Lactone-Based Biosensors

**DOI:** 10.3390/md15030053

**Published:** 2017-02-23

**Authors:** Kumar Saurav, Valeria Costantino, Vittorio Venturi, Laura Steindler

**Affiliations:** 1 Department of Marine Biology, Leon H. Charney School of Marine Sciences, University of Haifa, Mt. Carmel, 31905 Haifa, Israel; lsteindler@univ.haifa.ac.il; 2 The NeaNat Group, Dipartimento di Farmacia, Università degli Studi di Napoli Federico II, Via D. Montesano 49, 80131 Napoli, Italy; costanti@unina.it; 3 Bacteriology Group, International Centre for Genetic Engineering & Biotechnology, Padriciano 99, 34149 Trieste, Italy; venturi@icgeb.org

**Keywords:** quorum-sensing inhibitors, quorum quenching, marine natural products, antimicrobial resistance, *N*-acyl homoserine lactones

## Abstract

Marine natural products with antibiotic activity have been a rich source of drug discovery; however, the emergence of antibiotic-resistant bacterial strains has turned attention towards the discovery of alternative innovative strategies to combat pathogens. In many pathogenic bacteria, the expression of virulence factors is under the regulation of quorum sensing (QS). QS inhibitors (QSIs) present a promising alternative or potential synergistic treatment since they disrupt the signaling pathway used for intra- and interspecies coordination of expression of virulence factors. This review covers the set of molecules showing QSI activity that were isolated from marine organisms, including plants (algae), animals (sponges, cnidarians, and bryozoans), and microorganisms (bacteria, fungi, and cyanobacteria). The compounds found and the methods used for their isolation are the emphasis of this review.

## 1. Introduction

Overuse of antibiotics has led to a decrease in the effectiveness of the drugs currently available to combat life-threatening and debilitating diseases. As a survival mechanism, many pathogenic microbes have developed mechanisms for resisting the toxic effect of antimicrobials. Antimicrobial resistance can be encoded by “resistance genes” and susceptible strains can become resistant either through mutations in existing genes or by acquiring “resistance genes” from another organism that is already resistant [1,2,3]. There is an urgent need to discover new antimicrobial compounds and identify new methods for disease prevention and treatment [4]. Different factors can be important to avoid the emergence of new resistant traits: (a) understanding the basic concepts of infection and effective treatment; (b) keeping adequate infection control measures in hospitals to prevent the emergence of nosocomial resistant strains [5,6,7]; and (c) discovering new drugs and/or novel inhibitory pathways for fighting systemic infections [8].

**Figure 1:**
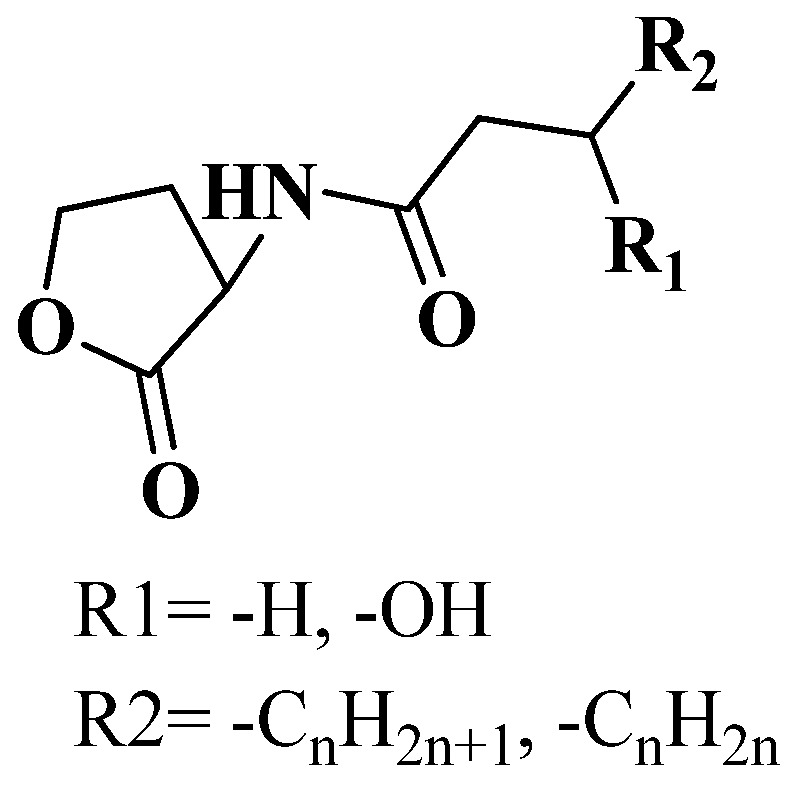
*N*-acyl homoserine lactones, found in many Gram-negative bacteria, vary by substitution at the C3 position (R1) and the length of the acyl chain (R2).

An increased understanding of the mechanism underlying bacterial pathogenesis and intercellular microbial communication has revealed potential alternative/complementary strategies to treat bacteria-mediated diseases [9,10]. Specifically, the strategy relates to interference of intercellular signaling to alter the pathogen’s ability to coordinate and invade their hosts, whether human, animal, or plant. Quorum sensing (QS) acts as a tool that synchronizes physiological activities of bacteria based on cell density and by means of genetic regulation [11]. In many cases, the responses elicited by QS signals contribute directly to pathogenesis through the coordinated production of virulence determinants, such as toxins, proteases, and other immune-evasive factors. Additionally, QS can contribute to behaviors that enable bacteria to resist antimicrobial compounds, such as biofilm development [12]. If the QS signal communication that coordinates these pathogenic behaviors is blocked, it is theorized that bacteria would lose their ability to mount an organized assault on the host and thus would be less able to form organized community structures that promote resistance to antibiotics [12,13,14]. Communication interference was not a human invention; rather, it exists naturally in the microbial world and is employed to gain an advantage over competitors [15]. Different types of QS communication systems rely on diverse small, secreted signaling molecules also known as autoinducers (AIs), which can act much like hormones in higher organisms, to initiate coordinated responses across a population. Autoinducers belong to different categories including, among others, the well-studied *N*-acylated homoserine lactones (AHLs), sometimes referred to as autoinducer-1 (AI-1), used by many Gram-negative bacteria (Figure 1); oligopeptide-based signals, used by Gram-positive bacteria; and a shared furanone-based system (autoinducer-2, AI-2), used by both Gram-negative and Gram-positive bacteria. There are also other QS signals that go beyond these classes, including *Pseudomonas* quinolone signal (PQS), diffusible signal factor (DSF), and autoinducer-3 (AI-3) [16]. 

Despite the presence of diverse quorum-sensing network architectures, the key steps of signal supply and signal response are conserved in any QS system [12,17]; hence, QS inhibitors (QSI) can be categorized into two functional classes: signal-supply inhibitors and signal-response inhibitors, depending on whether a drug inhibits the function of signal generation or the response [18]. In this review, we focus on interference with AHL-QS systems. Although QSI has been shown to be an effective anti-infective strategy in different host–microbe systems, recent studies have suggested that QSI may also pose selective pressure. For example, the standard laboratory culture conditions using nutrient-rich media may not correctly mimic natural infection conditions, and resistance to QSI drugs may develop or not based on the specific environment the pathogen is experiencing [19]. It has also been proposed that QS blockade may increase the prevalence of more virulent traits among nosocomial infections [20]. For example, genetic knockouts in *P. aeruginosa* QS signal response show that signal-blind cheats are able to exploit a protease that is produced by signal-responsive individuals during well-mixed growth in both in vitro and animal models [21,22]. Further, resistance to one well-characterized QSI compounds, the brominated furanone C-30, was shown to be accomplished by the overexpression of the MexAB–OprM efflux pump [23]. While these and other recent studies provide evidence that QSI, as it is presently understood by the scientific community, is not the magic and final solution to antibiotic resistance in microbial pathogens, they provide motivation for more investigations into QSI compounds and mechanisms of action that can lead in the future to an efficient use of QSI as therapeutic treatment. 

QSI metabolites, sometimes also referred to as quorum-quenching (QQ) compounds, have been recovered from extracts of marine algae (e.g., [24]), invertebrates (e.g., [25]), terrestrial plants (e.g., [26,27]) and bacteria (e.g., [28]). Also, the enzymatic inactivation of QS signals was reported in bacterial extracts (e.g., [29,30]), mammalian cells [31], and plants [27]. Although QS and QQ processes were both first discovered in marine organisms [32,33], information on QQ processes in the marine environment is scarce when compared to its terrestrial counterparts but some indirect evidence suggests QSI as a frequent phenomenon in the marine environment. Numerous reports are emerging that provide empirical data demonstrating QSI activity from various marine sources including sponges, algae, bryozoan, and coral-associated bacteria [16]. Various methods have been implemented to identify strains that produce QSI compounds, followed by lead molecule purification. QS biosensors, which are genetically modified strains that express reporter genes (e.g., *lacZ*, *gfp* or *luxCDABEG*) in response to the presence of specific QS signals [34], are also valuable tools for the identification of QSI molecules. Here, we provide the list of the most widely used AHL-based biosensors that were applied for bioassay-guided isolation and identification of QSI lead molecules from marine sources (Table 1). The aim of this review is to give an overview of marine-derived secondary metabolites with QSI activity that have been described to date using AHL-based biosensor assays. The compounds were categorized based on their source of isolation.

## 2. Bacterial AHL-Based Biosensors

An AHL-mediated QS system uses AHLs as signal molecules. AHLs consist of an invariant homoserine lactone (HSL) ring attached to an acyl chain with varying length (C_4_-C_20_) and various substituent (methylene, keto, or hydroxy groups) at the C_3_ position (Figure 1). AHLs are most commonly synthesized by members of the LuxI family of AHL synthases with a few exceptions such as *Vibrio harveyi* and *Vibrio fischeri*, which are synthesized by LuxM and AinS, respectively [52,53]. The identification of a large number of AHL QS systems was facilitated by several qualitative and quantitative methods to detect AHLs based on bacterial biosensors. These biosensors do not produce AHLs and contain a functional response-regulator (e.g., LuxR-type) protein cloned together with a cognate target promoter (often the promoter of the cognate AHL synthase gene), which positively regulates the transcription of a reporter gene (e.g., bioluminescence, β-galactosidase, green-fluorescent protein (*gfp*), and violacein pigment production) [34]. The same biosensors were later utilized also for the screening of QSI molecules. Selection of suitable biosensors plays a crucial role in screening and isolation of QSI compounds and depends on the type of study that is being carried out. The following section indicates some of the selective biosensors commonly employed for screening and identification of active QSI lead molecules based on their phenotypic expression.

### 2.1. Pigment-Based Biosensors

One of the most widely used biosensor in this category is based on *Chromobacterium violaceum*, a Gram-negative bacterium that produces the visible purple pigment violacein, whose production is under the regulation of the CviI/CviR QS system. McClean and collaborators developed an AHL-deficient and nonpigmented mutant of this species, the *C. violaceum* strain CV026, by mini-T5 mutagenesis in *cviI* and in a QS repressor locus (Sm^r^ mini-Tn*5* Hg^r^*cviI*::Tn*5xylE* Km^r^) [54]. This mutant will appear white unless provided with exogenous cognate AHL, which will result in the production of violacein and turn purple. Therefore, strain CV026 can be used as a biosensor to detect the presence of a range of AHLs, as well as being useful for screens of QSI molecules via the addition of exogenous AHLs and the detection of reduced violet pigmentation in the presence of QSI compounds in the tested extract. This pigment-based biosensor has been used both qualitatively and quantitatively for QSI compound searching; for example, using the overlaid well diffusion assay [54] or the violacein quantification assay in a 96-well plate [55]. CV026 was also previously used with Thin Layer Chromatography (TLC) for QSI screens, yet its detection limit for AHLs, C_4_-C_8_ acyl side chain length enables the search for QSI compounds that specifically affect the response to these signals [56]. Another *C. violaceum*-based mutant strain obtained through transposon insertion (Sm^r^ mini-Tn5 Hg^r^) of the wild strain *C. violaceum* ATCC 31532, and termed CV017, which results in the overproduction of violacein [57], has also been employed for bioactive guided isolation of QSIs (Table 1).

### 2.2. Bioluminescence-Based Biosensor

Bioluminescence is a common reporter activity used to quantify gene expression at high sensitivity and over a large dynamic range in real time and non-destructively [58]. The *lux* genes essential for luminescence are arranged in a single operon designated as *luxCDABE*. The *luxCDE* genes encode for a fatty acid reductase complex involved in synthesis of the fatty aldehyde substrate for the luminescence reaction catalyzed by the luciferase *luxAB* subunits [59]. Although all the *lux* genes isolated so far were derived from Gram-negative bacteria, their functional properties can vary between bacterial species of origin. For example, the *luxCDABE* operon from *Photorhabdus luminescens* permits a greater flexibility and ease of use in Gram-negative bacteria than the *luxAB* or *luxCDABE* systems derived from *Vibrio fischeri* or *Vibrio harveyi*, due to its wide temperature activity dependency [60]. Among others, the two most widely used bioluminescence-based QS reporters are plasmids pSB401 and pSB1075. Plasmid pSB401 was constructed using the *P. luminescens luxCDABE* operon controlled by the P*luxI* gene together with the *V. fischeri luxR* DNA fragment, and when transformed in *E. coli* it emits luminescence in response to the exogenous addition of AHLs with medium (C_6_-C_8_) acyl side chain length. LasR-based reporter plasmid pSB1075 contains the *lasR* gene, and the promoter of *lasI^−^* controlling the expression for reporter operon *luxCDABE* and emits luminescence in response to AHLs with long (≥C_10_) acyl side chain. Another plasmid, called pSB403, was constructed with the same arrangement as pSB401, and the advantage of being cloned into a wider host range replicon so it can be harbored in several other Gram-negative bacteria other than *E. coli* [58]. Swift et al. [61] constructed a similar biosensor (pSB536), sensitive to C_4_-AHL, using the *ahyR* AHL sensor/regulator of *Aeromonas hydrophyla* and the cognate *ahyI* gene promoter fused to *luxCDABE*. A quite different case is the bioluminescence-based reporter biosensor QSIS2, which was specifically developed for QSI assays. It is based on *lasI-rhlI* double mutant harboring the gene promoter of *lasB* controlling the expression of *sacB1*, encoding an AHL-induced killing system. The bacterial cell also harbors the plasmid pSU2007, which encodes the constitutively expressed *luxCDABE*, thus giving rise to bioluminescence expression in cases where the biosensor growth is rescued by the presence of a QSI molecule [62]. Another biosensor is the *V. harveyi* JMH 612 double mutant (*luxQ:cqs S_vh_*) enabling it to detect bioluminescence at very low cell density; it can thus be used for density-dependent bioluminescence assay [63].

QSI assays using bioluminescence-based biosensors can be visualized quantitatively/qualitatively by using bioluminescence microscopy or can be measured quantitatively using a luminometer. The general principle behind QSI assays is similar (with a few exceptions e.g., QSIS2) for the biosensors mentioned above. For a quantitative analysis, overnight cultures of these biosensors are inoculated in fresh medium at low OD (e.g., 0.01) and supplemented with the relevant cognate AHL to stimulate QS-induced expression of the reporter gene. QSI test extracts or purified molecules are added to the same biosensor cultures and kept for incubation. The bioluminescence is recorded over time (usually every 30 min for 1–16 h), and the production of bioluminescence is measured and compared to the same culture kept in the absence of the QSI test extract or purified compound (solvent of QSI extract/compound is added as a control).

### 2.3. gfp-Based Biosensors

The green fluorescent protein (GFP) of the jellyfish *Aequorea victoria* is a useful tool for non-invasive, real-time detection of gene expression at the single-cell level without the addition of any chemical substrates. The *gfp* gene encodes for a 27 kD monomer having a complex spectrum with a major excitation peak at 395 nm, and a minor peak at 475 nm [64]. Fluorescence of GFP producing strains can be easily quantified using, for example, 96-well plate assays. GFP-fluorescence for AHL-QS discovery can also be detected by microscopy and TLC assays. The GFP-based AHL sensor plasmid pKR-C12 contains a *lasB*-*gfp* (ASV) translational fusion together with a P*lac* constitutively expressed *lasR* gene, which encodes for the LasR receptor protein cloned in the broad host-range plasmid pBBR1MCS-5 [65]. Another GFP-based AHL sensor is the plasmid pAS-C8, which is based on the QS system of *Burkholderia cepacia* and contains P*cepI-gfp* (ASV) fusion together with the *cepR* regulator gene placed under control of P*lac*. pAS-C8 biosensor is most sensitive to C_8_-HSL [65,66]. These biosensors can be used for qualitative and quantitative screens, where epifluorescence microscope detects green fluorescent cells as early as 15 min after the addition of its cognate AHL [67]. Biosensor *E. coli* JB525 has been used for quantitative estimation of QSI molecules. This biosensor is composed by *E. coli* harboring the *gfp* plasmid pJBA132. The plasmid expresses the LuxR AHL receptor of *Vibrio fischeri*, and once activated by exogenously provided AHLs, LuxR induces the expression of the translational fusion P*luxI-gfp* (ASV) [67]. The QSIS3 system was constructed in host *E. coli* and is based on *Vibrio fischeri* LuxR QS system. The *npt* (kanamycin resistance gene) and *gfp* genes are controlled by the temperature-sensitive *cI* repressor, which in turn is regulated by QS through P*luxI*. In the presence of a QSI molecule, de-repression of antibiotic resistance leads to growth of the biosensor strain, which in turn produces GFP fluorescence, as the promotorless *gfp* gene is incorporated immediately downstream of the *npt* gene [62]. Hentzer et al. constructed a QS reporter system harboring the *lasB-gfp* (ASV) fusion in a *P. aeruginosa* PAO1 Tn5-Las background where the expression of unstable Gfp (ASV) is regulated by the QS-controlled P*lasB*. Upon reaching quorum, the biosensor produces fluorescence, yet in the presence of an exogenous QSI compound the decrease in fluorescence expression will be proportional to the concentration and efficacy of the QS inhibitor [68].

### 2.4. β-Galactosidase-Based Biosensor

β-galactosidase (β-gal) is a well-characterized bacterial enzyme containing one tetramer with a large subunit size of 1023 amino acids (the monomer is 116 kDa). β-gal can efficiently accelerate the hydrolysis of various β-galactosides, and has been used extensively as an endpoint control for assessing variability in reporter protein activity. The substrate 5-bromo-4-chloro-3-indolyl galactoside (X-Gal), a galactose sugar with a glycosidic linkage to an indolyl molecule, has been used widely as a reporter substrate. It remains colorless until the chromophore is linked to the galactose; however, upon hydrolysis in the presence of the enzyme β-gal, it produces a blue insoluble derivative, which is used in the reporter assay to visualize the activity [69]. Of all the AHL biosensors constructed using this reporter gene, the *Agrobacterium tumefaciens*-based sensor is particularly well suited and often used for TLC analysis and QS signals. *A. tumefaciens* strain NT1 carrying plasmid pZLR4 is one of the commonly used biosensors. NT1 does not harbor the Ti plasmid and accordingly does not produce native AHLs. Plasmid pZLR4 contains *tra*R and a fusion of *lacZ* (encoding for β-gal) to *traG*, which is expressed from a TraR-dependent promoter, thus producing a blue colony appearance in the presence of exogenous AHLs [70]. In contrast, a colorless colony appears in the presence of a QSI molecule in the extract. This sensor can also be used in overlay techniques, similarly to the *C. violaceum* CV026 biosensor (as previously described) [71]. Another *E. coli* plasmid sensor based on the β-gal reporter is pKDT17, which was constructed by adding the P*lac-lasR* sequence into plasmid pTS400, which contains a *lasB::lacZ* translational fusion. Rasmussen et al. developed a unique suicide-based reporter system, QSIS1, to identify QSI molecules. This system expresses the *luxR* gene and a translational fusion of P*luxI* with *phlA* (from *S. liquefaciens* MG1), the latter producing a toxic compound. When QS is active, the *phlA* is expressed, and the biosensor is killed. Accordingly, the system is used as a biosensor for QSI compounds, in the presence of such molecules, QS is inhibited, *phlA* is not expressed, and the QSIS1 is alive expressing *lacZ* and appearing as a blue zone surrounding the sample containing the QSI compounds [62].

## 3. Marine Microorganisms

Marine microbes are particularly attractive in terms of their potential application in pharmaceutical industry sector because they (a) have not been extensively exploited as their terrestrial counterparts; and (b) possess potent bioactive compounds in order to be effective in the diluted marine seawater environment [72]. It is estimated that less than 1% of potentially useful chemicals from marine environments has been screened so far, reflecting the very low percentage of microbial products originating from marine microorganisms [73]. The exploration of marine microbial secondary metabolites has led to the discovery of numerous biologically active compounds that possess antibiotic, antitumor, and other pharmacological activities which are currently being used for the treatment of various diseases in humans, animals, and plants [74,75]. Natural products with antibiotic activity that derive from bacteria or fungi are almost always products of secondary metabolic pathways [76].

### 3.1. QSI from Marine Bacteria

**Figure 2:**
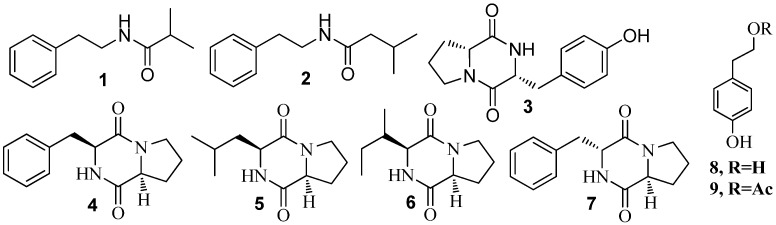
Structures of phenethylamides (**1**–**2**), cyclic dipeptides (**3**–**7**), tyrosol (**8**) and tyrosol acetate (**9**).

*Halobacillus salinus* C42 isolated from a seagrass sample collected in 2002 from Point Judith Salt Pond, South Kingstown, RI led to the isolation of two phenethylamide metabolites, *N*-(2′-phenylethyl) isobutyramide (**1**) and 2,3-methyl-*N*-(2′-phenylethyl)-butyramide (**2**) (Figure 2), with potential to inhibit QS. Theses metabolites were quantitatively assessed for QS inhibition using *Chromobacterium violaceum* CV026, *Vibrio harveyi* BB120 and *Escherichia coli* JB525 at a non-inhibitory concentration below 200 µg/mL. These compounds behaved as antagonists of bacterial QS by competing with *N*-acyl homoserine lactones for receptor binding [35]. From the same location, the analysis of another Gram-positive bacterium, *Bacillus cereus* D28, yielded a cyclic dipeptide, cyclo(l-Pro-l-Tyr) (**3**) (Figure 2), using bioassay guided fractionation. QSI activity was measured using disk diffusion assays with *V. harveyi* BB120 and *C. violaceum* ATCC 12472 [28].

*Marinobacter* sp. SK-3, isolated from a microbial mat sample collected from wadi Muqshin in southeastern Oman, resulted in the isolation of four diketopiperazines (DKPs), cyclo(l-Pro-l-Phe) (**4**), cyclo(l-Pro-l-Leu) (**5**), cyclo(l-Pro-l-isoLeu) (**6**), and cyclo(l-Pro-d-Phe) (**7**) (Figure 2), with QSI properties when screened using *C. violaceum* CV017 and *Escherichia coli*-based QS reporters (pSB401 and pSB1075). **4** and **6** inhibited QS-dependent production of violacein by *C. violaceum* CV017. **4**, **5**, and **6** reduced the QS-dependent luminescence of the reporter *E. coli* pSB401 induced by 3-oxo-C_6_-HSL [39]. Cyclo(Pro-Leu) (**5**) was also identified in an active subfraction of the extract from *Staphylococcus saprophyticus* by GC-MS analysis with moderate QSI activity [77]. The chemical study of the organic extract of *Oceanobacillus profundus* isolated from the Caribbean soft coral *Antillogorgia elisabethae*, yielded compounds tyrosol (**8**) and tyrosol acetate (**9**) (Figure 2), as responsible for the QSI activity detected using *C. violaceum* ATCC 31532 [78].

### 3.2. QSI from Marine Fungi

**Figure 3:**
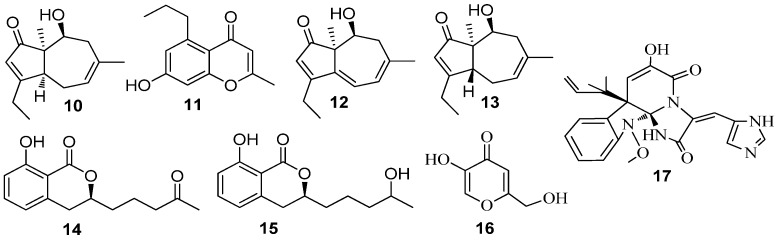
Structures of Aculene E (**10**), Penicitor B (**11**), aculene C (**12**), aculene D (**13**), aspergillumarins A (**14**), aspergillumarins B (**15**), kojic acid (**16**) and meleagrin (**17**).

Marine fungal strains are potent producers of polyketide-derived alkaloids, terpenes, peptides, and mixed biosynthesis compounds that represent chemical groups of secondary metabolites produced by fungi [79]. A fermentation broth of *Penicillium* sp. SCS-KFD08 associated with marine animal *Sipunculus nudus* from Haikou Bay, China resulted in the isolation of six QSI active compounds (**10**–**15**) (Figure 3), using *C. violaceum* CV026 as a biosensor. These compounds exhibited strong QSI activity at a dosage of 50 µg/well using the well diffusion assay. At a non-inhibitory concentration, compounds **10** and **15** strongly reduced the violacein production by up to 49.1% and 45.5%, respectively, at a concentration of 300 µM [36]. Kojic acid (**16**) (Figure 3), previously isolated from marine-derived *Alternaria* sp. from the surface of the marine green alga *Ulva pertusa* [80] inhibited QS-dependent violacein production of the reporter *C. violaceum* CV017 at MIC of 239.25 µM and reduced the bioluminescence of reporter *E. coli* pSB401 induced by 3-oxo-C_6_-HSL at concentrations above 36 μM [40]. Meleagrin (**17**) (Figure 3), a known bacterial enoyl-acyl carrier protein reductase (FabI) inhibitor from the marine fungus *Penicillum chrysogenium* [81], inhibited QS of the bacterial reporter *C. violaceum* CV017 with MIC of 138.42 µM [40].

### 3.3. QSI from Marine Cyanobacteria

**Figure 4:**
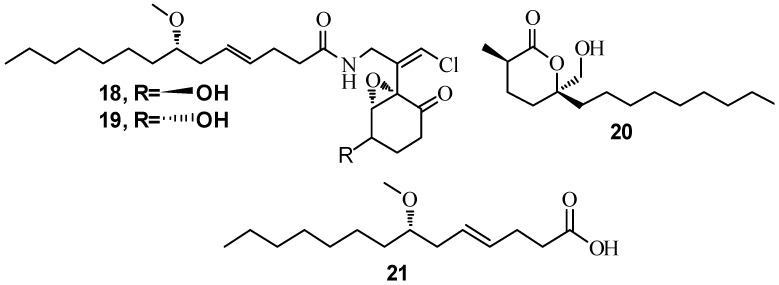
Structures of 8-epi-malyngamide C (**18**), malyngamide C (**19**), malyngolide (**20**) and lyngbyoic acid (**21**).

**Figure 5:**
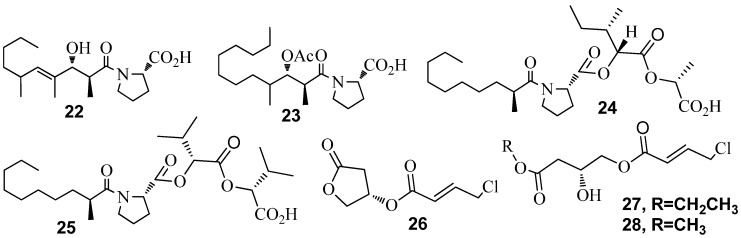
Structures of tumonoic acids (**22**–**25**) and honaucins A–C (**26**–**28**).

Cyanobacteria are an ancient group of organisms well known for their prolific production of bioactive natural products [82,83]. Since 2013, there has been an increase in the number of new metabolites reported from cyanobacteria [84]. Metabolites from the well-studied genus *Lyngbya* have been examined for production of QSI inhibitory compounds in addition to many other bioactive screens. Extracts of *L. majuscula* collected at various sites in Florida yielded 8-epi-malyngamide C (**18**), malyngamide C (**19**) and malyngolide (**20**) and a new small cyclopropane-containing fatty acid, lyngbyoic acid (**21**) (Figure 4). Compounds **18** and **19** were able to reduce 3-oxo-C_12_-HSL induced signaling in a *LasR*-based QS reporter (pSB1075) at a concentration of 10 µM [85]. **20** was identified as a QS inhibitor using the pSB1075 reporter with an EC_50_ value of 12.2 ± 1.6 µM. It was also found to inhibit *LasR*-regulated phenotypic production of elastase by *P. aeruginosa* PAO1 with EC_50_ = 10.6 ± 1.8 µM [41]. Compound **21** antagonized 3-oxo-C_12_-HSL induced bioluminescence of the pSB1075-based sensor with an IC_50_ of approximately 100 µM. **21** also reduced production of QS regulated pyocyanin and elastase in wild-type *P. aeruginosa* [24]. *Blennothrix cantharidosmum* collected at Duke of York Island in Papua New Guinea in 2005 resulted in the isolation of tumonoic acids (**22**–**25**) (Figure 5), which inhibited bioluminescence in wild-type strain *Vibrio harveyi*, with compound **23** having the strongest activity, IC_50_ of 62 μM [84].

*Leptolyngbya crossbyana* led to the isolation of three γ-butyrolactones, honaucins A–C (**26**–**28**) (Figure 5), from corals collected on the Hawaiian coast. Honaucins A–C presented a dose-dependent QSI by reducing bioluminescence expression in *Vibrio harveyi* BB120, with IC_50_ values of 5.6, 17.6, and 14.6 μM, respectively. **26**–**28** also exhibited potent inhibition of lipopolysaccharide-stimulated nitric oxide production in the murine macrophage cell line RAW264.7 [86].

**Figure 6:**
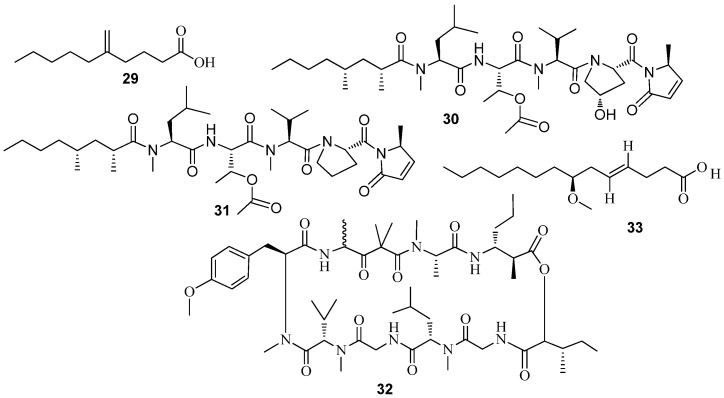
Structures of Pitinoic acid A (**29**) microcolins A (**30**) and B (**31**) and a peptide lyngbyastatin 3 (**32**) and lyngbic acid (**33**).

Pitinoic acid A (**29**) (Figure 6), was isolated from a Guamanian cyanobacterium as an inhibitor of QS in *P. aeruginosa* and significantly reduced the transcript levels of *lasB* and the pyocyanin biosynthetic member *phzG1* at 1 mM and 100 μM, respectively. Additionally, it also reduced levels of *LasB* and pyocyanin in the culture supernatants as evaluated by an enzymatic assay for *LasB* and quantitative evaluation using UV absorbance for pyocyanin at 1 mM [87]. Two lipopeptides, microcolins A (**30**) and B (**31**) and a peptide lyngbyastatin 3 (**32**) (Figure 6), from *Lyngbya majuscula* inhibited the QS of the bacterial reporter *C. violaceum* CV017 with an MIC of 15.23 µM, 43.21 µM, and 12 µM, respectively [40]. Recently, lyngbic acid (**33**) (Figure 6), has been isolated from a polymicrobial disease consortium dominated by the filamentous cyanobacterium *Roseofilum reptotaenium* with strong QSI activity against *Vibrio harveyi*-based QS reporters [48].

## 4. QSI from Marine Sponges

**Figure 7:**
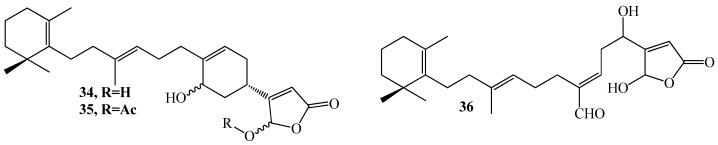
Structures of manoalide (**34**), manoalide monoacetate (**35**), and secomanoalide (**36**).

Sponges (phylum Porifera) are sessile marine filter feeders evolutionarily considered as the oldest animals and the richest natural product source. Up to 2013, approximately 4851 discovered compounds contributing to nearly 30% of all marine natural products belong to marine sponges [88]. In 2014 alone, 283 new compound structures reported from sponges enabled the phylum to regain its position as the dominant source of new bioactive metabolites [75]. Compounds range from terpene skeletons, mixed polyketide-peptide biogenesis molecules to complex carbohydrate-based metabolites [89,90,91,92,93]. Many of these metabolites have been tested for their possible role as lead compounds in drug research. Extracts from *Luffariella variabilis* led to the isolation of three sesterterpene metabolites, manoalide (**34**), manoalide monoacetate (**35**), and secomanoalide (**36**) (Figure 7), with strong QS inhibition properties based on the QS reporter system QSIS1 and QSIS2. In addition, the activities for compound **34**–**36** were also quantitatively tested using biosensor *P. aeruginosa* PAO1 Tn5-Las with IC_50_ values of 0.658 mM, 1.123 mM, and 1.110 mM, respectively [25]. 

**Figure 8:**
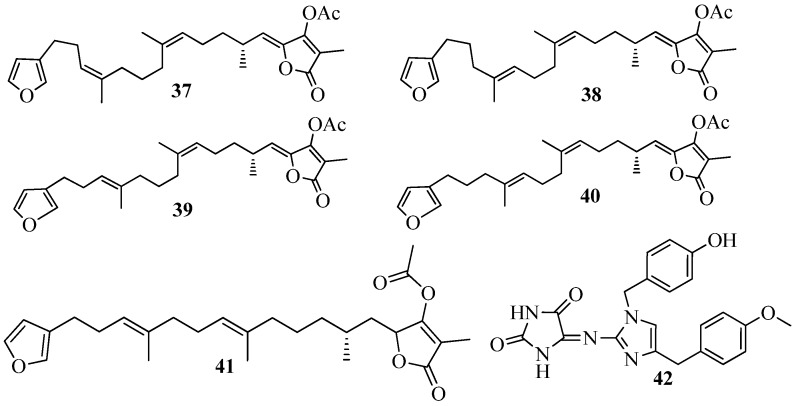
Structures of furanosesterterpenes (**37**–**41**) and isonaamine D (**42**).

QSI screening studies on marine invertebrates (39 extracts) including 26 sponges, seven soft corals, five algae, and one zooanthid collected in the Colombian Caribbean Sea and the Brazilian Coasts led to the isolation of five furanosesterterpenes (**37**–**41**) (Figure 8), from the sponge *Ircinia felix*. Compound (7*Z*,13*Z*,18*R*,20*Z*)-felixinin acetate (**37**), (8*Z*,13*Z*,18*R*,20*Z*)-strobilinin acetate (**38**), (7*E*,13*Z*,18*R*,20*Z*)-felixinin acetate (**39**) and (8*E*,13*Z*,18*R*,20*Z*)-strobilinin acetate (**40**) was isolated with distinct configuration along with compound (7*E*,12*E*,18*R*,20*Z*)-variabilin acetate (**41**) using *C. violaceum* biosensor [43]. These compounds were previously reported from *I. felix* based on their antimicrobial properties [94,95]. Similarly, Saurav et al. [96] screened marine sponges from the Mediterranean and Red Sea for QSI activity using the QSIS1 biosensor and *C. violaceum* CV026 systems and observed, QSI as a common phenomenon within half of the sponge species tested. Four sponge species extracts were observed to inhibit QS activity; more precisely, two Red Sea sponges, *Suberites clavatus* and *Negombata magnifica*, and two Mediterranean Sea species, *Ircinia variabilis* and *Sarcotragus* sp. However, the lead molecules responsible for this activity have not been characterized but it has been reported that sponge genera *Ircinia* sp. and *Sarcotragus* sp., both belonging to the family Irciniidae, are a potential source of QSI active lead molecules [97]. In addition, the investigation of the crude extract of *Leucetta chagosensis* led to the isolation of isonaamine D (**42**) (Figure 8), with mild but dose-dependent activity on the *Vibrio harveyi* JMH 612 bioreporter [49].

**Figure 9:**
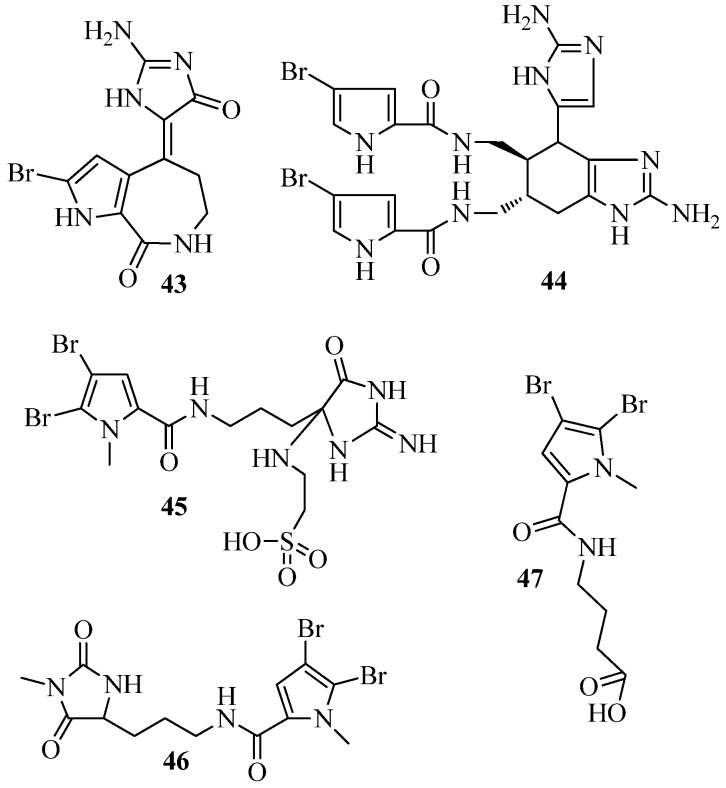
Structures of Hymenialdisin (**43**), ageliferin (**44**), mauritamide B (**45**), midpacamide (**46**) and butanoic acid (**47**).

Dobretsov et al. [40] screened a large set of natural products for QSI activity. Five compounds (**43**–**47**) (Figure 9), deriving from various sponges were able to inhibit QS of *C. violaceum* CV017. Hymenialdisin (**43**), an alkaloid originally isolated from the sponge *Axinella verrucosa* [98] inhibited QS dependent luminescence in reporter strain pSB401 and pSB1075 induced by 3-oxo-C_6_-HSL and 3-oxo-C_12_-HSL, respectively. Several other alkaloids, ageliferin (**44**) isolated from *Agelas conifer*, mauritamide B (**45**) isolated from *A. nakamurai*, midpacamide (**46**), isolated from *A. mauritama* [99] and 4-(4,5-dibromo-1-methyl-1*H*-pyrrole-2-carboxamido) butanoic acid (**47**) isolated from *Agelas* sp. [100] inhibited QS of the bacterial reporter *C. violaceum* CV017 with an MIC of 11.29, 36.76, 458.61 and 271.73 µM, respectively.

**Figure 10:**
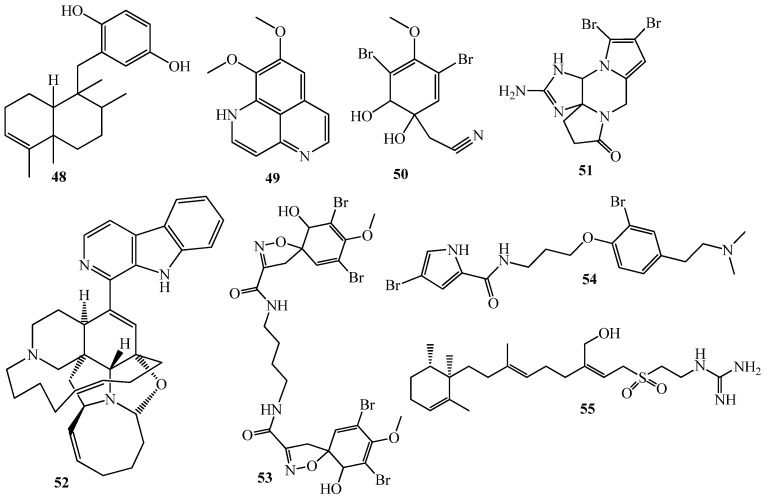
Structures of (+)avarol (**48**) and seven alkaloids (**49**–**55**).

On the other hand, one terpene, (+)avarol (**48**) and seven alkaloids (**49**–**55**) (Figure 10), from distinct marine sponges inhibited the QS of *C. violaceum* CV017, along with antibiotic properties [40,101].

## 5. QSI from Marine Algae

**Figure 11:**
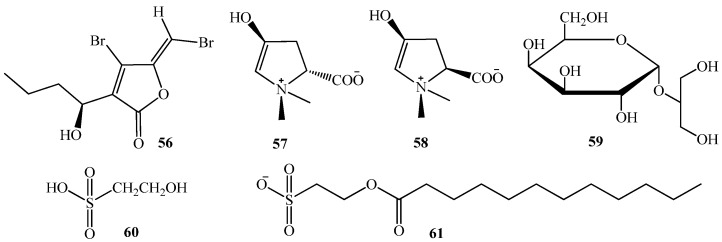
Structures of halogenated furanones, (**56**), betonicine (**57**), *cis*-betonicine (**58**), floridoside (**59**), and isethionic acid (**60**) and 2-dodecanoyloxyethanesulfonate (**61**).

Marine algae act as a major food source in marine environments and produce a variety of compounds that are beneficial to human health [102,103]. These compounds include polyunsaturated fatty acids and carotenoids as well as other compounds with antibiotic and antifungal activity [104,105,106]. Several polyunsaturated fatty acids are also being studied in relation to their potential anticancer activity and treatment of the symptoms of cystic fibrosis [75,107,108]. The first QS antagonist compounds discovered from macro-algal source were isolated from *D. pulchra* and *Ahnfeltiopsis flabelliformis*. They include halogenated furanones, (**56**) [44,109], betonicine (**57**), *cis*-betonicine (**58**), floridoside (**59**), and isethionic acid (**60**) (Figure 11). They display an inhibition activity on QS mechanisms mediated by C_8_-AHL lactone and the TraR transcriptional activator protein [38,50,51,110]. ICR-FT/MS analysis revealed the presence of 2-dodecanoyloxyethanesulfonate (**61**) (Figure 11), in *Asparagopsis taxiformis*, as the lead molecule responsible for QSI activity based on *Serratia liquefaciens* MG44 and *C. violaceum* CV026 bioassays. This is in line with previous reports showing that sulfur-containing synthetic AHL-analogues can inhibit QS, such as the case of *N*-(propylsulfanylacetyl)-l-homoserine lactone, and *N*-(pentylsulfanylacetyl)-l-homoserine lactone [37,111].

## 6. QSI from Cnidarians

**Figure 12:**
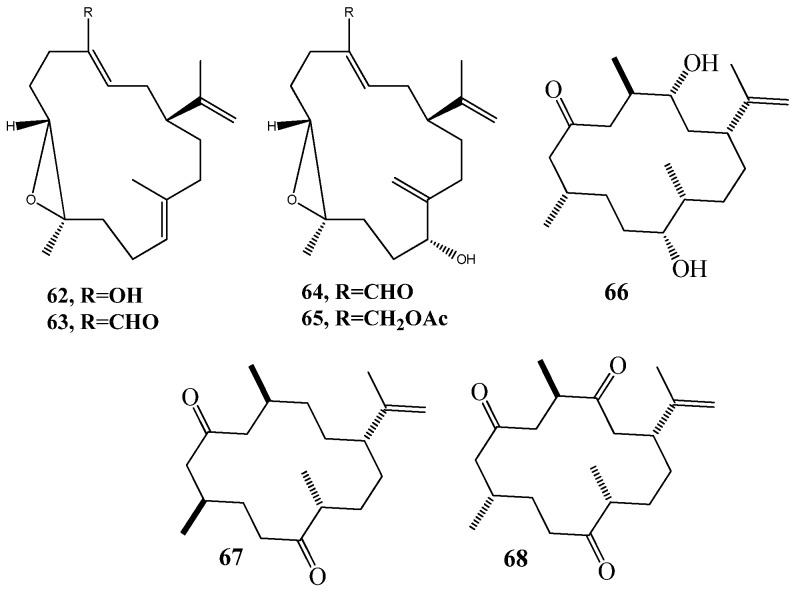
Structures of cembranoids (**62**–**68**).

Cnidaria is a large, diverse, and ecologically important group of marine invertebrates (including jellyfish, sea anemones, and corals) that is characterized by specialized cells called cnidocytes or nematocytes, utilized for prey capture. Over 3000 natural products have been described from this phylum, mostly in the last two decades [112,113]. The ability of cnidarians to produce powerful toxins and venoms has also been well documented [114]. In terms of QSI compounds, four cembranoids (**62**–**65**) (Figure 12), were isolated from octocoral *Eunicea knighti* with QSI property. QSI assays were carried out using *P. putida* IsoF wild strain, *E. coli* pSB401, and *C. violaceum* (ATCC 31532) biosensor [45,46]. Another three cembranoid epimers at C-8 (**66**–**68**) (Figure 12), were isolated from the Colombian Caribbean octocoral *Pseudoplexaura flagellosa* based on inhibition of the production of violacein pigment of *C. violaceum* (ATCC 31532) biosensor [47].

## 7. QSI from Bryozoa

**Figure 13:**
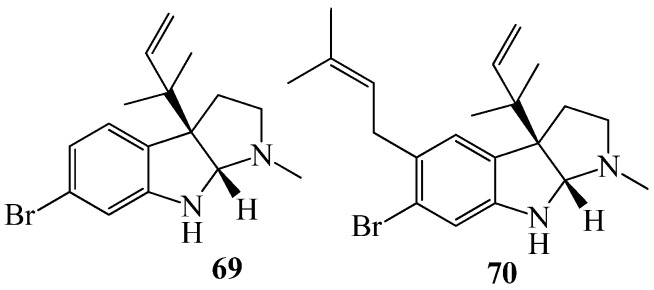
Structures of two brominated alkaloids (**69**, **70**).

Bryozoans, also known as sea mats or sea mosses, are found in both freshwater and marine environments. Natural products and biological activity have been reported from all the bryozoan orders constituting the marine clade [115]. By using the biosensors *P. putida* (pKR-C12), *P. putida* (pAS-C8), and *E. coli* (pSB403) the antagonistic effect on *N*-acyl-homoserine lactone-dependent QS systems was investigated on metabolites from North Sea bryozoan *Flustra foliacea,* leading to the isolation of two QSI compounds, **69** and **70**, (Figure 13), active at concentrations of 20 μg/mL [42].

## 8. Concluding Remarks

The utilization of QSI could be a promising strategy for antivirulence therapy as it is less prone to the development of resistance. Moreover, natural heritable variation in QS genes and their expression level may result in differences in fitness under conditions that are different from those in standard laboratory, and can thus pose selective pressure on bacteria to develop resistance. To date more than 70 marine-derived QSI lead molecules with diverse molecular entities have been discovered using AHL-mediated biosensor, and many more compounds are expected to be discovered in the near future. It was evident from previous reports that several alkaloids from sponges exhibit QSI properties and hence it is noteworthy to screen previously isolated alkaloids from sponges. For example, *Neopetrosia carbonaria*, a rich producer of quinolinic type alkaloids and other nitrogen-containing compounds with cytotoxic and antimicrobial activity [116,117], could also possess QSI properties. Similarly, various acridine-type polar alkaloids were isolated from *Xestospongia* cf. *carbonaria* and *Xestospongia* cf. *exigua* with anticancer activity [118]. Consequently, it could be hypothesized that these alkaloids might possess QSI activity of interest; however, it has not yet been demonstrated. 

## Figures and Tables

**Table 1:** AHL-based biosensors used for the identification of active QSI molecules from the sea.

Strain/Plasmid	QS System	Reporter System	Detection Range	References
*Chromobacterium violaceum* CV026	CviI/R	Violacein pigment	C_6_-HSL, 3-oxo-C_6_-HSL, C_8_-HSL, 3-oxo-C_8_-HSL	[35,36,37,38]
*Chromobacterium violaceum* CV017	CviI/R	Violacein pigment	3-oxo-C_6_-HSL, C_8_-HSL, 3-oxo-C_8_-HSL	[39,40,41]
*Escherichia coli* pSB403	LuxI/R (*V. fisheri*)	*luxCDABE*	3-oxo-C_6_-HSL, C_6_-HSL, 3-oxo-C_8_-HSL, C_8_-HSL	[37,42]
*Escherichia coli* pSB536	AhyI/R (*A. hydrophyla*)	*luxCDABE*	C_4_-HSL	[24]
*Escherichia coli* pSB401	LuxI/R (*V. fisheri*)	*luxCDABE*	3-oxo-C_6_-HSL, C_6_-HSL, 3-oxo-C_8_-HSL, C_8_-HSL	[39,40,43,44,45,46,47]
*Escherichia coli* pSB1075	LasI/R (*P. aeruginosa*)	*luxCDABE*	3-oxo-C_12_-HSL, 3-oxo-C_10_-HSL, C_12_-HSL	[39,40,41]
QSIS2	LasI/R (*P. aeruginosa*)	*luxCDABE*	3-oxo-C_12_-HSL, 3-oxo-C_10_-HSL, C_12_-HSL	[25]
*Vibrio harveyi* JMH 612	LuxPQ (*Vibrio harveyi*)	*luxQ*	3-OH-C_4_-HSL	[48,49]
*Agrobacterium tumifaciencens* pZLR4	TraI/R (*A. tumefaciens*)	β-galactosidase	All 3-oxo-HSLs	[41,50,51]
*Escherichia coli* pKDT17	LasI/R (*P. aeruginosa*)	β-galactosidase	3-oxo-C_12_-HSL, C_12_-HSL, C_10_-HSL, 3-oxo-C_10_-HSL	
QSIS1	LuxI/R (*V. fisheri*)	β-galactosidase	3-oxo-C_6_-HSL, C_6_-HSL, C_8_-HSL, C_10_-HSL	[25]
pAS-C8	CepI/R (*B. cepacia*)	*gfp*	C_8_-HSL	[42]
pKR-C12	LasI/R (*P. aeruginosa*)	*gfp*	3-oxo-C_12_-HSL, 3-oxo-C_10_-HSL	[42]
*Escherichia coli* JB525	LuxI/R (*V. fisheri*)	*gfp*	3-oxo-C_6_-HSL, C_6_-HSL, C_8_-HSL, C_10_-HSL	[35]
QSIS3	LuxI/R (*V. fisheri*)	*gfp*	3-oxo-C_6_-HSL, C_6_-HSL, C_8_-HSL, C_10_-HSL	[25]
Tn5-Las	LasI/R (*P. aeruginosa*)	*gfp*	3-oxo-C_12_-HSL, 3-oxo-C_10_-HSL	[25]
